# Supplementary material for: Raising Suicide in Medical Appointments—Barriers and Facilitators Experienced by Young Adults and GPs: A Mixed-Methods Systematic Review
Source: Int J Environ Res Public Health. 2023 Jan 1;20(1):822. doi: 10.3390/ijerph20010822 (PMC9820164; doi:10.3390/ijerph20010822)
Supplement: Supplementary file 1 [file ijerph-20-00822-s001.zip › Supplementary File S1.pdf]

S1 – Search results

Search protocol and illustrative search strategy

Suicide related terms: suic* OR self-harm OR NSSI OR non-suicidal self-injury
Young adult terms: Young adult OR young adults
General Practitioner terms: GP OR general practitioner OR primary care physician

Scopus search [Article title, Abstract, Keywords]

GP *OR* "general practitioner" *OR* "primary care physician"
AND suic* *OR* "self harm" OR NSSI OR "non-suicidal self-injury"
AND "young adult" *OR* "young adults"

Search conducted 19/1/22 – results 262. Last search 1/6/22 – 4 new records, 0 relevant.

Additional search conducted on 12 June with term "primary health care" added to above search. 165 new records – 1 relevant (Obando Medina et al., 2014 study identified in hand searches).

Web of Science [All fields]

GP *OR* "general practitioner" *OR* "primary care physician"
AND suic* *OR* "self harm" OR NSSI OR "non-suicidal self-injury"
AND "young adult" *OR* "young adults"

Search conducted 19/1/22 – results 60. Last search 1/6/22 – 2 new records, 0 relevant.

PsycINFO [Any Field]

GP *OR* "general practitioner" *OR* "primary care physician"
AND suic* *OR* "self harm" OR NSSI OR "non-suicidal self-injury"
AND "young adult" *OR* "young adults"

Search conducted 19/1/22 – results 45. Last search 1/6/22 – 2 new records, 0 relevant.

CINAHL Complete [All fields]

GP *OR* "general practitioner" *OR* "primary care physician"
AND suic* *OR* "self harm" OR NSSI OR "non-suicidal self-injury"
AND "young adult" *OR* "young adults"

Search conducted 19/1/22 – results 51. Last search 1/6/22 – 1 new records, 0 relevant
