# Supplementary material for: Raising Suicide in Medical Appointments—Barriers and Facilitators Experienced by Young Adults and GPs: A Mixed-Methods Systematic Review
Source: Int J Environ Res Public Health. 2023 Jan 1;20(1):822. doi: 10.3390/ijerph20010822 (PMC9820164; doi:10.3390/ijerph20010822)
Supplement: Supplementary file 1 [file ijerph-20-00822-s001.zip › Supplementary Table S2.pdf]

Table S2. Qualitative assessment of studies

#	Study	JBI Tool	Score %	Reasons for score below maximum
Qualitative studies				
1	Bailey et al. 2019 [35]	Qual.	80%	No acknowledgement of researcher influence on the research; nor statement on cultural or theoretical position.
2	Bellairs-Walsh et al. 2020 [40]	Qual.	100%	
3	Bellairs-Walsh et al. 2021 [41]	Qual.	100%	
4	Farr et al. 2021 [36]	Qual.	90%	No acknowledgement of researcher influence on research.
5	Michail & Tait 2016 [37]	Qual.	90%	No acknowledgement of cultural or theoretical position.
6	Mughal et al. 2021 [38]	Qual.	100%	
7	Obando Medina et al. 2014 [30]	Qual.	60%	Philosophical perspective guiding methodology is not stated.<br>No acknowledgement of researcher influence on the research; nor statement on cultural or theoretical position.
Quantitative studies				
8	Beckinsale et al. 2001 [42]	Quan.	63%	Survey instrument utilised not tested for construct validity. Possible confounding factors not acknowledged.
9	Michail et al. 2017 [39]	Quan.	88%	Survey instrument utilised not tested for construct validity

Qual. JBI (Joanna Briggs Institute) Critical appraisal checklist for qualitative research. Lockwood, Munn, & Porritt (2015). % score based upon number of questions to which there was a "Yes" answer out of possible ten questions.

Quan. JBI Critical Appraisal checklist for analytical cross-sectional studies. Moola et al. (2020). % score based upon number of questions to which there was a "Yes" answer out of a possible eight questions.
